# A Mediterranean-Style Diet with Lean Beef Lowers Blood Pressure and Improves Vascular Function: Secondary Outcomes from a Randomized Crossover Trial

**DOI:** 10.1016/j.cdnut.2025.104573

**Published:** 2025-02-22

**Authors:** Jennifer A Fleming, Kristina S Petersen, Penny M Kris-Etherton, David J Baer

**Affiliations:** 1 Department of Nutritional Sciences, The Pennsylvania State University, University Park, PA, United States; 2 USDA/ARS/BHNRC Food Components and Health Lab, Beltsville, MD, United States

**Keywords:** Mediterranean diet, lean beef, blood pressure, central blood pressure, arterial stiffness, cardiovascular disease

## Abstract

**Background:**

The Mediterranean (MED) dietary pattern improves cardiovascular disease (CVD) risk factors. Increased central systolic blood pressure and arterial stiffness are independent predictors of CVD. The effect of a MED diet on these measures of vascular health has not been investigated.

**Objectives:**

The aim was to evaluate the effects of a MED diet incorporating 0.5 oz./d (MED0.5), 2.5 oz./d (MED2.5) and 5.5 oz./d (MED5.5) of lean beef compared with an Average American diet (AAD) on vascular health [brachial and central blood pressure, pulse wave velocity (PWV), and augmentation index].

**Methods:**

A multicenter, 4-period randomized, crossover, controlled-feeding study was conducted at Penn State University and USDA, Beltsville. In random sequence order, participants consumed each test diet for 4 wk. Vascular outcomes were assessed at baseline and the end of each diet period. Linear mixed models were used for analyses.

**Results:**

Between-diet differences were observed for peripheral and central blood pressure as well as PWV (*P <* 0.05). PWV was lower following MED0.5 [−0.24 m/s; 95% confidence interval (CI): −0.44, −0.04] and MED2.5 (−0.27 m/s; 95% CI: −0.47, −0.07) compared with the AAD; PWV was nominally lower after the MED5.5 compared with the AAD (−0.20 m/s; 95% CI: −0.40, 0.003; *P* = 0.055). Central systolic blood pressure was lower following the MED0.5 (−3.24 mmHg; 95% CI: −5.22, −1.27) and MED2.5 (−2.93 mmHg; 95% CI: −4.91, −0.96) compared with the AAD. A similar pattern was observed for central diastolic pressure. Brachial systolic and diastolic pressure were lower following all 3 MED diets compared with the AAD (*P <* 0.05).

**Conclusions:**

Compared with an AAD, MED diets containing 0.5 and 2.5 oz./d of lean beef improved brachial and central systolic and diastolic blood pressure and arterial stiffness. Our findings suggest that a MED diet with ≤5.5 oz./d of lean beef does not adversely affect vascular function.

This trial was registered at clinicaltrials.gov as NCT02723617.

## Introduction

Cardiovascular disease (CVD) is the number one cause of global disease burden accounting for ∼30% of all deaths [1]. Given that poor diet quality is a significant contributor to cardiovascular morbidity and mortality [2], a healthy dietary pattern is recommended to promote cardiovascular health [3]. Heart-healthy dietary patterns emphasize fruits, vegetables, whole grains, and liquid plant oils, and include healthy protein sources (mostly plant-based; regular intake of fish and seafood; low-fat or fat-free dairy products; and if meat or poultry is desired, lean cuts and unprocessed forms in moderation are recommended). Although heart-healthy dietary patterns limit lean unprocessed red meat, evidence from clinical trials shows that the intake of lean unprocessed red meat as part of healthy dietary patterns does not adversely affect lipids/lipoproteins or peripheral blood pressure (BP) [[4], [5], [6]]. To our knowledge, previous research has not examined the effect of healthy diets incorporating lean unprocessed beef on central BP or arterial stiffness.

Traditional measures of brachial systolic and diastolic BP (SBP and DBP) do not reflect the pressure load in the large conduit arteries because the arterial pulse is modified as it travels away from the heart to the periphery. On the basis of anatomical proximity, central pulse pressure (PP) more closely reflects the pulsatile stress experienced by the heart, and as such, is considered a better predictor of CVD events [1,7,8]. Similarly, increased carotid-femoral pulse wave velocity (PWV) [[9], [10], [11], [12], [13], [14]] is an independent predictor of adverse cardiovascular events including mortality and may explain some of the residual CVD risk observed in individuals with well-controlled hypertension [15,16].

Strong and consistent evidence shows Mediterranean (MED)-style diets decrease CVD risk [17]. Typically, MED diets are low in red meat. In the United States, red meat (including beef) is a popular food. Increasingly, the importance of customizing dietary choices to reflect personal preferences is recognized to promote sustained adherence to a healthy dietary pattern [18]. Therefore, although current dietary guidance consistently recommends limiting red meat, more clarity is needed about the amount of lean unprocessed red meat that can be incorporated into healthy dietary patterns that promote cardiovascular health. The present study was designed to address this gap. The aim was to evaluate the effect of including lean beef [14, 71, 156 g/d/2000 kcal (0.5, 2.5, 5.5 oz./d/2000 kcal)] as part of a healthy MED-style diet on brachial and central BP, PWV and augmentation index (AI) compared with an average American diet (AAD) containing ∼71 g (2.5 oz.) beef/d/2000 kcal. It was hypothesized that the MED diets containing lean beef would lower BP and PWV regardless of beef amount included compared with the AAD. These findings will inform recommendations for heart-healthy diets that meet personal preferences for the inclusion of lean beef.

## Methods

### Study design

The methods used for this study and the primary endpoint results, including lipids and lipoproteins, have been reported in detail elsewhere [4]. This article reports the results of prespecified secondary outcomes, including brachial and central BP, PWV, and AI. Briefly, a 4-period, randomized, crossover, controlled-feeding study was conducted at 2 centers: Penn State University and USDA-Beltsville Human Nutrition Research Center. The fully controlled, weight-maintenance dietary feeding intervention provided a fixed macronutrient composition that varied only between the MED diets (41% fat, 42% carbohydrate, and 17% protein) and the AAD (33% fat, 52% carbohydrate, and 15% protein) (Table 1). Energy requirements were calculated using the Harris–Benedict equation and adjusted for self-reported exercise. The amount of lean beef consumed was based on the calculated energy requirements of the participants, with a 2000 kcal diet providing 14, 71, and 156 g (0.5, 2.5 and 5.5 oz.)/d for the MED0.5, MED2.5, and MED5.5, respectively. Participants were randomly assigned to 1 of 12 diet sequences to ensure that diets were assigned in a balanced order. An independent USDA staff member generated the block randomization code using an orthogonal Latin-square design with 5 blocks (5 replicates) and 12 sequences per block. After randomization, the study coordinators, investigators, and statisticians were blinded for the purposes of outcome assessment and statistical analysis. The Institutional Review Board at the Pennsylvania State University and MedStar Health Research Institute (for Beltsville Human Nutrition Research Center) approved the study protocol before the initiation of the study, and all participants provided written informed consent. The trial is registered at clinicaltrials.gov (Identifier: NCT02723617).

**TABLE 1:** Nutrient targets of the test diets.

Nutrient	AAD	MED0.5	MED2.5	MED5.5
Total fat (% E)	33	41	41	41
SFA (% E)	12	8	8	8
MUFA (% E)	13	25.5	25.5	25.5
PUFA (% E)	8	7.5	7.5	7.5
Carbohydrate (% E)	52	42	42	42
Protein (% E)	15	17	17	17
Cholesterol (mg)	<300	<300	<300	<300
Sodium (mg)	∼3500	<2300	<2300	<2300
Beef (oz./d)	≈ 2.5 oz.	0.5 oz.	2.5 oz.	5.5 oz.
ALA (g)	1.5	1.5	1.5	1.5
Marine n-3 (g)	<0.1	0.5	0.5	0.5

Values were determined using Food Processor (ESHA Research).

Abbreviations: % E, percentage of total energy; AAD, average American diet; ALA, alpha-linolenic acid; MED, Mediterranean-style eating pattern used in the study; MED0.5, MED diet with 14 g (0.5 oz.)/d of lean beef; MED2.5, MED diet with 71 g (2.5 oz.)/d of lean beef; MED5.5, MED diet with 156 g (5.5 oz.)/d of lean beef based on a 2000 kcal diet.

Participants consumed each diet for 4 wk with a washout period of ≥1 wk, in which participants resumed their self-selected diet between diet periods. Participants’ adherence during the trial was monitored with daily questionnaires regarding the intake of study and nonstudy foods/beverages, medications, exercise, and general wellness. Participants were instructed to consume only the foods provided and to limit their consumption of alcohol (≤ 2 drinks/wk) and noncaloric caffeinated beverages (<1180 mL/d or <40 oz.). Intake of any nonstudy foods was recorded, and any remaining study foods were returned to the site. Adherence was estimated to be >90% based on participant reports and study food(s) not eaten. Participants had the option to consume their meals on-site (Monday–Friday) or have their meals prepared and packed for off-site consumption (Penn State) or, at the Beltsville site, participants consumed their breakfast and dinner on-site (Monday–Friday) and consumed their lunch off-site. At both sites, weekend meals and snacks were packaged for off-site consumption.

### Study participants

**FIGURE 1:**
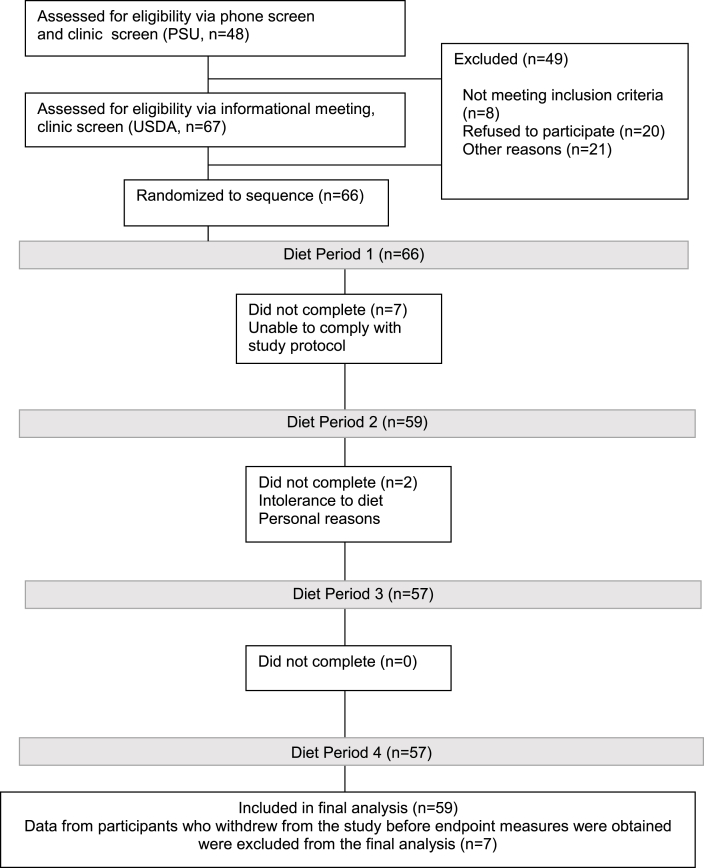
CONSORT diagram showing the flow of participants through each stage of the randomized trial.

Participants (target *n* = 60; 30 per site) were generally healthy nonsmoking males and females with a BMI >20 and <40 kg/m^2^, aged 30–70 y that were recruited between October 2016 and November 2017 from the State College, PA and Beltsville, MD areas. Individuals prescribed BP-lowering medications were eligible if their BP was <160/100 mmHg and they had been on a stable medication dose for ≥6 mo. Participant recruitment for both sites is presented in Figure 1. Detailed cohort characteristics and sample size determination were described previously [14].

### Clinical visits

Vascular endpoints were collected on the first of 2 consecutive clinic visits at baseline (start of study) and at the end of each 4-wk diet period. Testing was not performed at the start of diet periods 2, 3, and 4. For the 48 h before each clinical assessment, participants were asked to refrain from consuming alcohol and using anti-inflammatory medications. For the 24 h before each clinical assessment, participants were asked not to engage in vigorous exercise, and not to consume any food or drink (except water) for the 12 h before their visit.

### Vascular health

#### Resting peripheral BP

After a 5-min seated rest period, brachial artery SBPs and DBPs were measured in the left arm using an automated BP cuff (Penn State: Omron HEM-705CP; Omron Healthcare; USDA: Datascope Accutorr Plus Monitor; Mindray). At each time point, 3 measures were taken, following JNC 7 BP guidelines [19], with 1 min between each reading. The last 2 results were averaged and used for analysis.

#### Pulse wave analysis: central (aortic) BP and AI

The SphygmoCor XCEL system (AtCor Medical) was used to assess central SBP, central DBP, AI (adjusted to a heart rate of 75 beats/min), and PWV. Before measurements, participants were fitted with the correct size BP cuff, and these cuffs were used consistently for the remainder of the study. Trained research personnel performed all measurements in a quiet, temperature-controlled room. Central pressures were derived from the measured brachial artery pressure wave using a validated generalized transfer function. AI was obtained from the synthesized central pressure wave and calculated as the ratio between augmented pressure and central PP [AI = (P2−P1)/(Ps−Pd), where P1 is first shoulder of systolic pressure, P2 is second shoulder of systolic, Ps is peak systolic pressure, and Pd is end-diastolic pressure]. The value was standardized to a heart rate of 75 beats/min to correct for the independent inverse effect of heart rate on the pulse wave form [20].

#### PWV

Carotid-femoral PWV was measured using the SphygmoCor XCEL system (AtCor Medical). Carotid and femoral arterial pressure waveforms were measured simultaneously via applanation tonometry of the right common carotid artery and a BP cuff placed on the right femoral artery. Distance measurements were taken from the sternal notch to the carotid artery, sternal notch to the top of the femoral cuff, and femoral artery to the top of the femoral cuff. The SphygmoCor System automatically calculates carotid-femoral PWV by dividing the measured aortic distance (distal–proximal) by the average measured time delay between the initial upstroke of the corresponding carotid and femoral waveforms. The measure was performed while the participant was in a supine position.

### Statistical analysis

Sample size calculations conducted for the primary outcome, LDL cholesterol, showed that 60 participants (*n* = 30 per site) would provide 90% power to detect an effect size of 0.46 (α = 0.05) as previously reported [4]. All statistical analyses were performed using SAS 9.4 (Statistical Analyses System). Residuals were assessed for normality by visual inspection of distributions (histograms and stem and leaf plots), skewness value, and Shapiro-Wilk *P* value. The analytic plan was designed a priori and described a mixed-effect model for analysis of the data for repeated measurements (PROC MIXED). All data were analyzed in a manner consistent with intention-to-treat principles. Available data from every randomly assigned participant were included in the analyses. Data from participants who withdrew from the study were included when endpoint measures were obtained. The mixed models procedure does not perform listwise deletion and preserves the degrees of freedom; thus, it allows for inclusion of participants with ≥1 missing data points in the analyses. Mixed models were used to test the main effects of diet. For all models, baseline value, sex, randomization sequence, period, and site were included as covariates, subject nested within randomization sequence was included as a repeated effect, and diet was a fixed effect. A treatment by diet order analysis was conducted to test for carryover effects. Model covariance structures were based on optimizing fit statistics (evaluated as lowest Bayesian Information Criterion). For each variable, the following analyses were performed—a comparison of means following each diet, the change from baseline following each diet (calculated by subtracting baseline values from endpoint values) as well a comparison of the change from baseline between the test diets. Values are reported as least square means ± SEM. Where a significant main effect of diet was detected, post hoc testing was conducted, and the Tukey–Kramer method was used to adjust for multiple comparisons. The results of the post hoc testing are presented as least squares mean difference and Tukey–Kramer adjusted 95% confidence intervals (CI) and *P* values (*P <* 0.05).

## Results

Sixty-six individuals were enrolled in the study. A total of 9 individuals withdrew from the study. Of those, 7 withdrew before completing the first diet period because of an inability to comply with study procedures. The remaining 2 withdrew after diet period one. Data from individuals who did not complete ≥1 full diet period(s) were not included in the analyses (*n* = 7). Statistical testing showed no evidence of carryover effects. The participants (*n* = 59; age: 49 ± 1.6 y; BMI: 27 ± 0.5 kg/m^2^) were generally healthy with multiple CVD risk markers within recommended ranges at the start of the study (Table 2). All participants maintained their baseline weight throughout the study.

**TABLE 2:** Characteristics of study participants at baseline (*n* = 59).

	Mean ± SD
Age (y)	49 ± 12
Males:females (*n*)	28:31
BMI (kg/m^2^)	27 ± 4
TC (mg/dL)	193 ± 37
LDL cholesterol (mg/dL)	110 ± 27
HDL cholesterol (mg/dL)	55 ± 15
TG (mg/dL)	105 ± 60
Glucose (mg/dL)	99 ± 9
SBP (mmHg)	117 ± 14
DBP (mmHg)	77 ± 9

Baseline values were measured before consuming any study food.

Abbreviations: DBP, diastolic blood pressure; SBP, systolic blood pressure; TC, total cholesterol; TG, triglycerides.

### Peripheral BP

Brachial SBP was lower following the MED0.5 (mean difference −3.42 mmHg; 95% CI: −5.99, −0.85; *P* = 0.004), MED2.5 (−3.14 mmHg; 95% CI: −5.72, −0.56; *P* = 0.01), and MED5.5 (−2.66 mmHg; 95% CI: −5.24, −0.08; *P* = 0.04) compared with the AAD. Brachial DBP was lower following the MED0.5 (−2.78 mmHg; 95% CI: −4.69, −0.86; *P* = 0.001), MED2.5 (−2.74 mmHg; 95% CI: −4.66, −0.82; *P* = 0.002) and MED5.5 (−1.95 mmHg; 95% CI: −3.87, −0.03; *P* = 0.046) compared with the AAD. No differences in brachial SBP or DBP were observed between the 3 MED diets (*P* > 0.05) (Table 3).

**TABLE 3:** Central and brachial blood pressures at baseline and after 4 wk of consuming each test diet^1^.

Endpoint	Baseline^2^	AAD	MED0.5	MED2.5	MED5.5	*P* value
Central SBP (mmHg)	113 ± 1.68	111 ± 0.68^a^	108 ± 0.68^b^	108 ± 0.69^b^	109 ± 0.69^a,b^	<0.001
Central DBP (mmHg)	75 ± 0.96	74 ± 0.55^a^	71 ± 0.55^b^	71 ± 0.55^b^	72 ± 0.55^a,b^	<0.001
Brachial SBP (mmHg)	117 ± 1.74	115 ± 0.99^a^	112 ± 0.99^b^	112 ± 1.00^b^	113 ± 1.00^b^	0.002
Brachial DBP (mmHg)	77 ± 1.17	76 ± 0.63^a^	73 ± 0.63^b^	73 ± 0.64^b^	74 ± 0.64^b^	0.001

Abbreviations: AAD, average American diet (2.5 oz beef/d); DBP, diastolic blood pressure; MED, Mediterranean-style eating pattern used in the study; MED0.5, MED diet with 14 g (0.5 oz.)/d of lean beef; MED2.5, MED diet with 71 g (2.5 oz.)/d of lean beef; MED5.5, MED diet with 156 g (5.5 oz.)/d of lean beef based on a 2000 kcal diet; SBP, systolic blood pressure.

1 All values are least squares means ± SEMs (*n* = 59) unless otherwise stated. The MIXED procedure (version 9.4; SAS Institute Inc.) was used to test the effects of diet on each outcome measure. The *P* value represents the main effect of diet. When a main diet effect was detected, post hoc tests were conducted to assess pairwise between-diet differences and adjusted for multiple comparisons using the Tukey–Kramer method. Values in the same row with different superscript letters are significantly different (Tukey-adjusted *P* < 0.05).

2 Data are presented as arithmetic mean ± SEMs.

**FIGURE 2:**
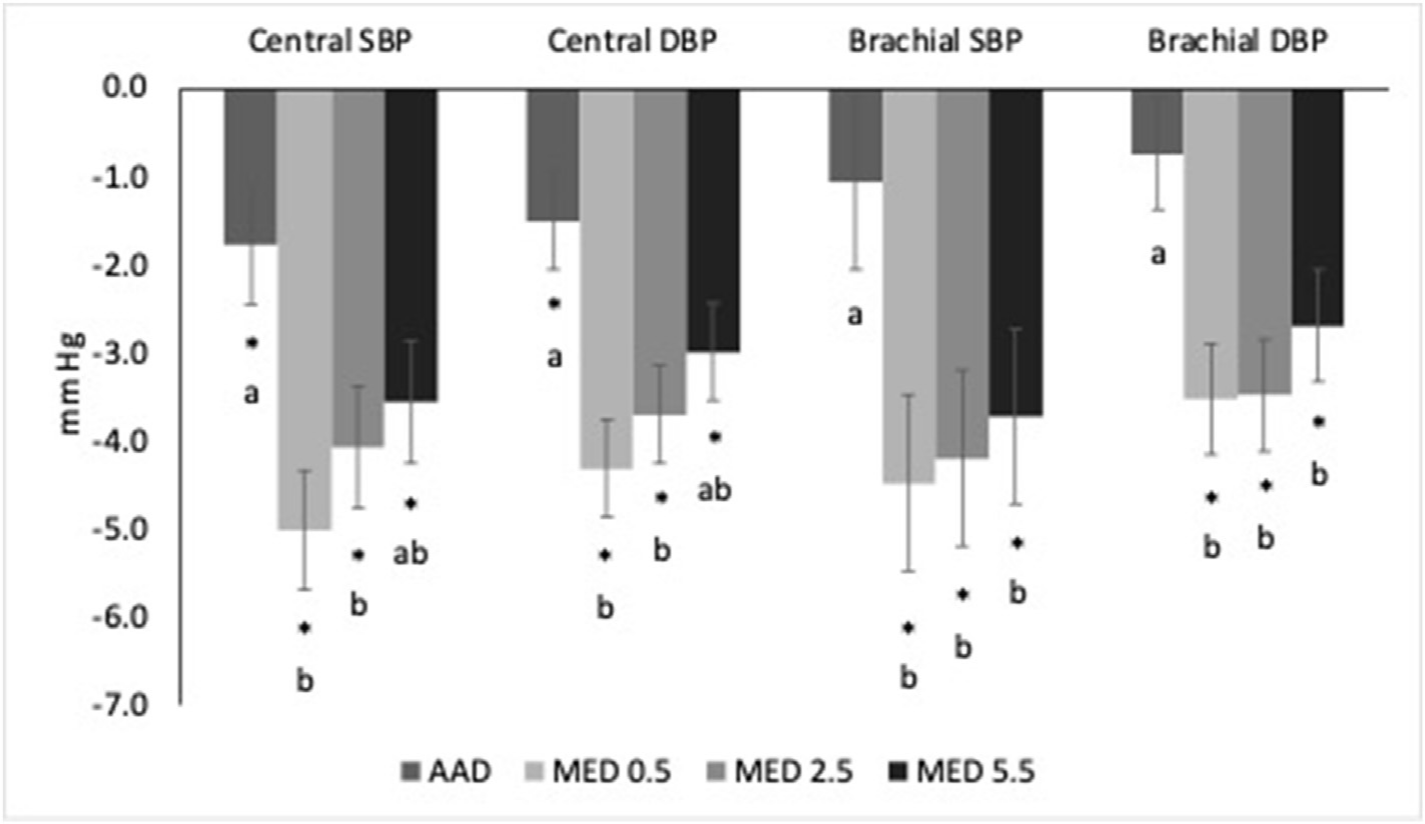
Change from baseline in central and brachial blood pressure following 4 wk of consuming each test diet. Data presented as least squares mean change (±SEM) from baseline (*n* = 59). The MIXED procedure in SAS (version 9.4; SAS Institute Inc.) was used to test for within- and between-diet effects. Where the main effect for diet was statistically significant, post hoc testing was conducted and values with different letters are significantly different (Tukey-adjusted *P* < 0.05). ∗Significantly different from baseline, *P* < 0.05. AAD, average American diet; DBP, diastolic blood pressure; MED, Mediterranean-style eating pattern used in the study; MED0.5, MED diet with 14 g (0.5 oz.)/d of lean beef; MED2.5, MED diet with 71 g (2.5 oz.)/d of lean beef; MED5.5, MED diet with 156 g (5.5 oz.)/d of lean beef based on a 2000 kcal diet; SBP, systolic blood pressure.

Brachial SBP was decreased from baseline to a greater extent following the MED0.5 (−3.42 mmHg; 95% CI: −5.99, −0.85; *P* = 0.004), MED2.5 (−3.14 mmHg; 95% CI: −5.72, −0.56; *P* = 0.01), and MED5.5 (−2.66 mmHg; 95% CI: −5.24, −0.08; *P* = 0.04) compared with the AAD. The decrease in brachial DBP from baseline was greater following the MED0.5 (−2.78 mmHg; 95% CI: −4.69, −0.86; *P* = 0.001), MED2.5 (−2.74 mmHg; 95% CI: −4.66, −0.82; *P* = 0.002), and MED5.5 (−1.95 mmHg; 95% CI: −3.87, −0.03; *P* = 0.046) compared with the AAD. For both brachial SBP and DBP, no differences were observed between the 3 MED diets (*P* > 0.05) (Figure 2).

### Central BP

Central SBP and DBP were decreased in all diet groups compared with baseline (Figure 2). Central SBP was lower following the MED0.5 (−3.24 mmHg; 95% CI: −5.22, −1.27; *P <* 0.001) and MED2.5 (−2.93 mmHg; 95% CI: −4.91, −0.96; *P* = 0.001) compared with the AAD. Central SBP tended to be lower following the MED5.5 compared with the AAD (−1.80 mmHg; 95% CI: −3.77, 0.18; *P* = 0.09). Central DBP was lower following the MED0.5 (−2.81 mmHg; 95% CI: −4.34, −1.27; *P <* 0.001) and MED2.5 (−2.19 mmHg; 95% CI: −3.73, −0.66; *P* = 0.002) compared with the AAD. Central DBP tended to be lower after the MED5.5 compared with the AAD (−1.49; 95% CI: −3.03, 0.05; *P* = 0.06). There were no differences in central SBP or DBP between the 3 MED diets (Table 3).

All diets lowered central SBP and DBP compared with baseline (Figure 2). Central SBP and DBP reductions from baseline were greater following MED0.5 (systolic: −3.24 mmHg; 95% CI: −5.22, −1.27; *P <* 0.001; diastolic: −2.81 mmHg; 95% CI: −4.34, −1.27; *P <* 0.001) and MED2.5 (−2.93 mmHg; 95% CI: −4.91, −0.96; *P* = 0.001; diastolic: −2.19 mmHg; 95% CI: −3.73, −0.66; *P* = 0.002) compared with the AAD. Central SBP and DBP reductions from baseline tended to be lower following the MED5.5 compared with the AAD (systolic: −1.80 mmHg; 95% CI: −3.77, −0.18; *P =* 0.09; diastolic: −1.49 mmHg; 95% CI: −3.03, 0.05; *P =* 0.06). No differences were observed between the MED diets (*P >* 0.05).

### Arterial stiffness

**FIGURE 3:**
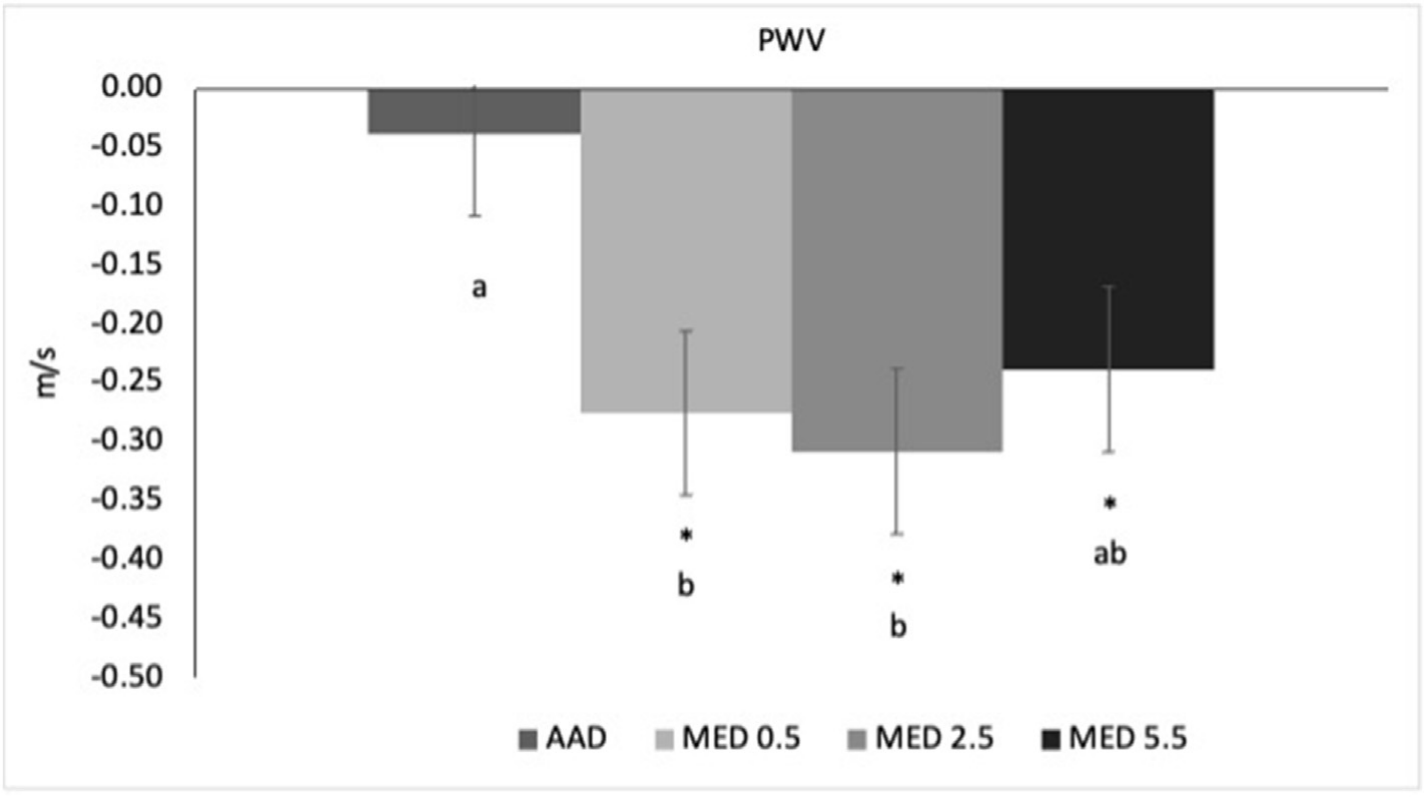
Change from baseline in PWV after 4 wk of consuming each test diet. Data presented as least squares means change (±SEM) from baseline (*n* = 59). The MIXED procedure in SAS (version 9.4; SAS Institute Inc.) was used to test for within- and between-diet effects. Where the main effect for diet was statistically significant, post hoc testing was conducted and values with different letters are significantly different (Tukey-adjusted *P* < 0.05). ∗Significantly different from baseline, *P* < 0.001. AAD, average American diet; MED, Mediterranean-style eating pattern used in the study; MED0.5, MED diet with 14 g (0.5 oz.)/d of lean beef; MED2.5, MED diet with 71 g (2.5 oz.)/d of lean beef; MED5.5, MED diet with 156 g (5.5 oz.)/d of lean beef based on a 2000 kcal diet; PWV, pulse wave velocity.

There were no differences among the diets for AI (Table 4). For PWV, no differences were detected among the 3 MED diets; however, PWV was significantly lower after the MED0.5 (−0.24 m/s; 95% CI: −0.44, −0.04; *P =* 0.01) and MED2.5 (−0.27 m/s; 95% CI: −0.47, −0.07; *P =* 0.004) compared with the AAD. PWV tended to be lower after the MED5.5 compared with the AAD (−0.20 m/s; 95% CI: −0.40, 0.003; *P =* 0.055). Compared with baseline, all MED diets improved PWV (*P <* 0.01); however, there was no change for AAD (Figure 3).

**TABLE 4:** PWV and augmentation index (AI at 75 bpm) at baseline and after 4 wk of consuming each test diet^1^.

Endpoint	Baseline^2^	AAD	MED0.5	MED2.5	MED5.5	*P* value
PWV (m/s)	7.1 ± 0.15	7.1 ± 0.1^a^	6.9 ± 0.1^b^	6.8 ± 0.1^b^	6.9 ± 0.1^ab^	0.003
AI at 75 bpm	22.2 ± 1.7	20.1 ± 1.1	19.0 ± 1.1	20.7 ± 1.1	21.0 ± 1.1	0.13

Abbreviations: AAD, average American diet (2.5 oz. beef/d); AI, augmentation index; MED, Mediterranean-style eating pattern used in the study; MED0.5, MED diet with 14 g (0.5 oz.)/d of lean beef; MED2.5, MED diet with 71 g (2.5 oz.)/d of lean beef; MED5.5, MED diet with 156 g (5.5 oz.)/d of lean beef based on a 2000 kcal diet; PWV, pulse wave velocity.

1 Data are presented as least squares means ± SEMs unless otherwise stated (*n* = 59). The MIXED procedure (version 9.4; SAS Institute Inc.) was used to test the effects of diet on each outcome measure. The *P* value represents the main effect of diet. When a main effect of diet was detected, post hoc tests were conducted to assess pairwise between-diet differences and adjusted for multiple comparisons using the Tukey–Kramer method. Values in the same row with different superscript letters are significantly different (Tukey-adjusted *P* < 0.05).

2 Data are presented as arithmetic mean ± SEMs.

## Discussion

A healthy dietary pattern is recommended for the prevention of CVD [21]. This is the first study, to our knowledge, to assess measures of central BP and arterial stiffness following a traditional MED diet as well as 2 MED diets containing higher quantities of lean unprocessed beef. In this study, both a traditional MED diet, containing 0.5 oz./d lean beef and a MED diet containing amounts approximately equivalent to United States (2.5 oz./d) intake at the time the study was designed significantly improved central BP as well as arterial stiffness (as assessed by PWV) when compared with a typical American diet containing 2.5 oz./d red meat.

The MED diets provided in this study resulted in peripheral SBP reductions that are clinically relevant (−3.4, −3.1, and −2.7 mmHg for the MED0.5, MED2.5, and MED5.5, respectively, compared with the AAD *P <* 0.05) despite the inclusion of participants with normal BP. There is a linear relationship between BP and incident CVD and therefore BP lowering even in the normal BP range is considered cardioprotective [22]. The effects we observed are similar to those reported in the original Dietary Approaches to Stop Hypertension (DASH) trial, whereby the DASH diet lowered SBP by 3.5 mm Hg in adults without hypertension compared with an AAD [23]. Our findings are also consistent with previous research [24] demonstrating that inclusion of lean beef (5.4 oz./d) in a DASH diet yielded similar reductions in SBP (−4.2 mmHg) compared with a traditional DASH diet (−2.8 mm Hg). For comparison, a 4 mmHg reduction in BP meets the United States Food and Drug Administration's requirement for antihypertensive drug approval [25].

Expanding on previous research, this study included PWV as a measure of arterial stiffness, which provides greater insight into the potential functional mechanisms that may be contributing to the vascular health benefits associated with a MED dietary pattern. Our findings show that the inclusion of ≤5.5 oz./d of lean beef in a MED dietary pattern does not attenuate PWV improvements. This study provides evidence for another dietary pattern that can be consumed by individuals who enjoy consuming lean beef. On the basis of the recent trends in the United States population, the average person consumes ∼1.5 oz. of beef daily [26], whereas we found significant improvements when including 2.5 oz lean beef as part of a healthy dietary pattern. This suggests that guidance should focus on building healthy dietary patterns rather than restriction or elimination of specific foods such as lean, unprocessed beef. In support of the 2020–2025 Dietary Guidelines for Americans, strategies aimed at customizing dietary patterns to reflect personal preferences, cultural differences, and accessibility are needed [18]. Moreover, the 2025 Dietary Guidelines Advisory Report recommends reducing intake of red and processed meats, and specific guidance will be needed about how to do this in the context of achieving a healthy dietary pattern [27]. Importantly, the findings presented herein provide guidance about the quantities of lean beef that can be included in a healthy dietary pattern.

The null effect of the MED diets on AI despite a decrease in PWV could be a result of the dissociation often seen between PWV and AI [28]. A recent cross-sectional study showed an inverse relationship between adherence to a MED diet and AI, but no association with PVW [29]. These discordant findings are in part because AI is influenced by several factors, including heart rate, sex, height, and age and as a result does not always correlate well with PWV. Moreover, AI depends upon the timing of the reflected waves as well as the intensity of the reflected waves. Because reflection time was reduced, as indicated by the reduction in PWV, yet AI was not, it could be speculated that changes occurred in the larger central arteries, not the peripheral arteries, in response to the MED diets.

Despite the benefits of a highly controlled randomized crossover feeding trial, our study has limitations. It is possible that the reduction in certain antioxidant-rich foods (for example, extra virgin olive oil, nuts, fish, fruits, and vegetables) as a result of the increase in lean beef [4] could explain the lack of difference between the MED5.5 and AAD. Future studies should ensure that servings of these components remain consistent across all MED diets, and instead, amounts of cereals and grains are altered. In addition, the AAD provided in this study was likely higher in potassium and calcium-rich foods than are typically consumed by our study participants such that the quantities of these nutrients may have contributed to the improvements in central BP following the AAD, despite the higher levels of sodium. However, when studying dietary patterns, the effects of individual components cannot be isolated. In the future, assessment of participant’s habitual dietary intake at baseline may provide greater insight regarding the observed changes from the AAD. In addition, baseline values were collected only once at the start of the study; however, statistical analyses included a test for residual diet effects. No effect of treatment order was identified. Data from previous controlled-feeding studies, ≤8 wk in duration, have demonstrated that lipids and lipoproteins stabilize after 4 wk and clinically meaningful changes are detected in 4 wk [30,31]. With lipids/lipoproteins as our primary outcome, 4-wk treatment periods were deemed appropriate. For BP, the DASH diet was 8 wk, with maximum BP reduction observed after 2 wk [23]. However, there are limited data on the duration of change for other measures of vascular health. In designing the treatment diets, we chose to include a MED diet with the traditional amount of red meat (14 g/d); however, it is unclear how the results may have differed if a diet containing no red meat was examined.

A major strength of our study is the randomized controlled crossover design and low dropout rate (<15%). High levels of dietary compliance were achieved as verified by the daily and weekly monitoring forms [4]. To our knowledge, this is the first study to examine the effects of a MED dietary pattern containing 3 levels of lean red meat on multiple measures of vascular health in a United States population. The consistency of our results with other studies on the MED diet demonstrates our ability to replicate the dietary pattern in a United States population with high levels of dietary adherence. Although our population was predominantly white, they were generally healthy at baseline, which limited the comorbidities and medication use as factors in our results. Given the reductions in BP that were observed in this healthy population, future studies should include individuals with hypertension on antihypertensive drugs to assess changes in medication status. Moreover, given that the MED dietary pattern significantly improved vascular health in this low-risk population, it is likely that even greater benefits would be observed in a cohort with hypertension as was observed in the DASH trial [23].

In conclusion, consumption of a MED-style dietary pattern containing 0.5–5.5 oz./d of lean beef significantly lowered brachial SBP and DBP when compared with an AAD containing 2.5 oz./d of beef. Greater improvements in central SBP and DBP and arterial stiffness were observed after consumption of MED diets with lean beef in amounts ≤2.5 oz./d when compared with an AAD. These findings suggest that incorporation of lean beef into a healthy dietary pattern such as the MED diet does not adversely affect CVD risk and, in fact, improves vascular health. Thus, a MED diet with 0.5–2.5 oz./d of lean beef is an option for those who wish to include lean red meat as part of a healthy dietary pattern.

## Author contributions

The authors’ responsibilities were as follows – PMK-E, DJB, JAF: designed the research; JAF, DJB: responsible for participant recruitment and conducting the research; JAF KSP: performed the statistical analyses; JAF, KSP, PMK-E: wrote the manuscript with editorial assistance from DJB; and all authors: read and approved the final manuscript.

## Data availability

Data described in the manuscript, code book, and analytic code will be made available on reasonable request.

## Funding

This trial was funded by the National Cattlemen's Beef Association, a contractor to the Beef Checkoff. This study also was supported by the USDA, ARS and the Penn State Clinical and Translational Science Institute, Pennsylvania State University Clinical and Translational Science Award, and NIH/National Center for Advancing Translational Sciences grant no. UL1 TR002014. Financial supporters had no role in the design and conduct of the study, collection, analysis, and interpretation of data, or preparation, review, or approval of the manuscript.

## Conflict of interest

JAF received travel funds from the National Cattlemen’s Beef Association for giving presentations on this research. PMK-E and DJB received funding from the National Cattlemen's Beef Association for the research reported in this article. KSP has received grants from the National Cattlemen’s Beef Association to conduct other research projects. KSP has also received honoraria from the National Cattlemen’s Beef Association for consulting work unrelated to the research presented in this paper.
